# Antimicrobial peptides in patients with anorexia nervosa: comparison with healthy controls and the impact of weight gain

**DOI:** 10.1038/s41598-020-79302-1

**Published:** 2020-12-17

**Authors:** Marie-Christin Bendix, Michael Stephan, Mariel Nöhre, Wally Wünsch-Leiteritz, Hagen Schmidt, Gisa Tiegs, Jürgen Harder, Martina de Zwaan

**Affiliations:** 1 Department of Psychosomatic Medicine and Psychotherapy, Hannover Medical School, Carl-Neuberg-Strasse 1, 30625 Hannover, Germany; 2 Klinik Lüneburger Heide, Competence Center for Eating Disorders, Bad Bevensen, Germany; 3 Institute of Experimental Immunology and Hepatology, University Medical Center Hamburg-Eppendorf, Hamburg, Germany; 4 Department of Dermatology, University Hospital Schleswig-Holstein, Kiel, Germany

**Keywords:** Immunology, Psychology

## Abstract

Clinical observations show that patients with anorexia nervosa (AN) are surprisingly free from infectious diseases. There is evidence from studies in *Drosophila melanogaster* that starvation leads to an increased expression of antimicrobial peptides (AMPs). AMPs are part of the innate immune system and protect human surfaces from colonization with pathogenic bacteria, viruses and fungi. We compared the expression of AMPs between patients with AN and healthy controls (HC) and investigated the influence of weight gain. Using a standardized skin rinsing method, quantitative determination of the AMPs psoriasin and RNase 7 was carried out by ELISA. Even though non-significant, effect sizes revealed slightly higher AMP concentrations in HC. After a mean weight gain of 2.0 body mass index points, the concentration of psoriasin on the forehead of patients with AN increased significantly. We could not confirm our hypotheses of higher AMP concentrations in patients with AN that decrease after weight gain. On the contrary, weight gain seems to be associated with increasing AMP concentrations.

## Introduction

Clinical observations report that patients with anorexia nervosa (AN) are surprisingly free from infectious diseases^1^. They show low incidences for both viral and bacterial infections, even if the latter does not differ significantly from healthy controls^2^. This is remarkable because although AN is a subtype of malnutrition, individuals with AN do not suffer the same increased incidence of infection as found in protein malnutrition^3–5^. An altered immune system caused by protein malnutrition usually leads to an increased risk for infections^6^. It has been suggested that despite their severely malnourished conditions, patients with AN seem to be protected against infections^7^. Interestingly, during refeeding and weight gain, patients seem to lose their protection and infections frequently reoccur^7^.

Overall, it has been shown that there exist similarities but also differences between AN and primary malnutrition regarding the immune system. Most notably, various pro-inflammatory cytokines seem to be elevated in AN suggesting a pro-inflammatory state in AN that does not appear to be present in primary malnutrition. Moreover, it has been suggested that these immunological changes do not occur solely secondary to malnutrition but might represent a primary risk factor contributing to the pathogenesis of AN^4^. These changes per se cannot explain the lower incidence of infections in patients with AN and there might be other mechanisms explaining the lack of association between AN and infectious diseases.

In *Drosophila melanogaster (common fruit fly)*, starvation induces the activation of antimicrobial peptide (AMP) genes. Becker et al.^8^ showed that besides the infection-triggered pathway, the activation of AMP depends on the energy-status of the cells. This activation is regulated by the transcription factor *FOXO*. During starvation, this mechanism assures the production of AMP to maintain the defense system in cases of stress and energy deficiency. AMPs are part of the innate immune system and can be found in humans as well. They protect body surfaces like the skin from colonization with pathogenic bacteria, viruses and fungi^9^. Produced by epithelial cells and white blood cells, this most primitive part of immunity is widely distributed in plants and animals and is responsible for the first line of host defense^10,11^. AMPs have even been considered as therapeutic agents for the treatment of infectious diseases^12^.

Since the skin represents the major surface of humans to the environment^13^, effective defense mechanisms are required to control the growth and colonization of microbes on the skin surface. AMPs build a chemical barrier in addition to the mechanical barrier of the skin itself^14–16^. On human skin, psoriasin and RNase 7 are the most abundant AMPs and are constitutively produced by healthy skin keratinocytes and sebaceous gland cells (sebocytes)^10,16^. While psoriasin is primarily an *E. coli* killing factor, RNase 7 has broad spectrum antimicrobial activity^14^. Recently, it could be demonstrated that RNase 7 is involved in directly activating an antiviral immune response in human keratinocytes^17^. Besides the high constitutively released film of AMPs on human skin, studies have revealed several mechanisms of stimulation^9^. Elevated concentrations of psoriasin can be found after stimulation with the pro-inflammatory cytokines interleukin (IL)-1, -17, -22 and tumor necrosis factor (TNF)-α, after contact with various bacteria and after exposure to ultraviolet-B radiation^16^. Similarly, RNase 7 levels are increased after contact with the pro-inflammatory cytokines IL-17A, TNF-α, interferon-γ, ultraviolet-B radiation or bacteria^10,11,15^. According to recent studies, the simultaneous presence of pro-inflammatory cytokines seems to have a particularly strong impact on AMP induction^18^.

The expression of AMPs in patients with AN has not been investigated so far and the influence of body weight or nutrition on the production of AMPs in humans is still unclear. The low incidence of infectious diseases in patients with AN might be supported by higher levels of AMP production due to starvation and due to increased pro-inflammatory cytokines.

We investigated the concentrations of psoriasin and RNase 7 on the skin of patients with AN using enzyme-linked immunosorbent essay (ELISA). We compared the AMP concentrations of patients with AN to age- and sex-matched healthy controls (HC) and examined the trajectory of AMP concentrations after weight regain in patients with AN. In line with the findings in *Drosophila melanogaster* we expected to find higher concentrations in patients with AN compared to healthy controls and to detect a decrease in AMP production with weight gain.

## Material and methods

### Participants

Female patients with AN were recruited in two eating disorder inpatient facilities (Klinik Lüneburger Heide and Hannover Medical School). They were investigated twice: shortly after hospitalization and after a weight gain of at least one body mass index (BMI) point. To verify an increase in body cell mass of at least 2 kg, we used bioelectrical impedance analysis (BIACORPUS RX 4004 M, Medical Healthcare GmbH, Karlsruhe, Germany) to measure body composition prior to the second testing. Only patients with a BMI lower than 15 kg/m^2^ on admission were included. Thirty-six patients with AN were approached; three declined participation. Thus, baseline measurements at the time of admission to the hospital were conducted in 33 patients. One patient dropped out of the study, thus 32 patients were tested again after a weight regain of at least one BMI point. Patients met diagnostic criteria for AN according to DSM-5 criteria^19^. AN according to DSM-5 is characterized by (a) a restriction of energy intake leading to a significant low body weight, (b) an intense fear of gaining weight or becoming fat, and (c) an unduly influence of body weight or shape on self-worth. A severity specifier is based on body mass index with a BMI of less than 15 kg/m^2^ representing the most severe form.

For comparison 33 healthy age-matched, normal-weight female controls (HC) were recruited and were tested once. These randomly chosen women were required to have a BMI in the normal-weight range between 18.5 and 25.0 kg/m^2^ and to be free of prior and current mental disorders.

The inclusion criteria required every study participant to be older than 16 years, to be non-smoker and to be visually skin-healthy on the sampling points. Participants had to be free of inflammatory disease and immunosuppressive drugs.

This study was approved by the ethics committee of the Hannover Medical School (3209-2016). All participants and their legal guardians, if necessary, gave their written informed consent prior to study inclusion. All research was performed in accordance with relevant guidelines and regulations.

### The antimicrobial peptides (AMPs)

This study focused on determining the AMPs psoriasin and RNase 7. These are the two most abundant AMPs on human skin surface.

### Sampling and sample procedure

The sample extraction was always conducted in the same room at the same time in the morning to avoid a putative influence of the circadian rhythm. Prior to the sampling, the participants were informed about the preparations required. They should not wash the sampling points nor use any cosmetics, creams or make-up in the morning of testing. Also physical exercise in the morning of the investigation should be avoided since physical exercise has shown to increase expression levels of several AMPs^20^. All patients were assessed by the same researcher to reduce inter-examiner error.

**Figure 1:**
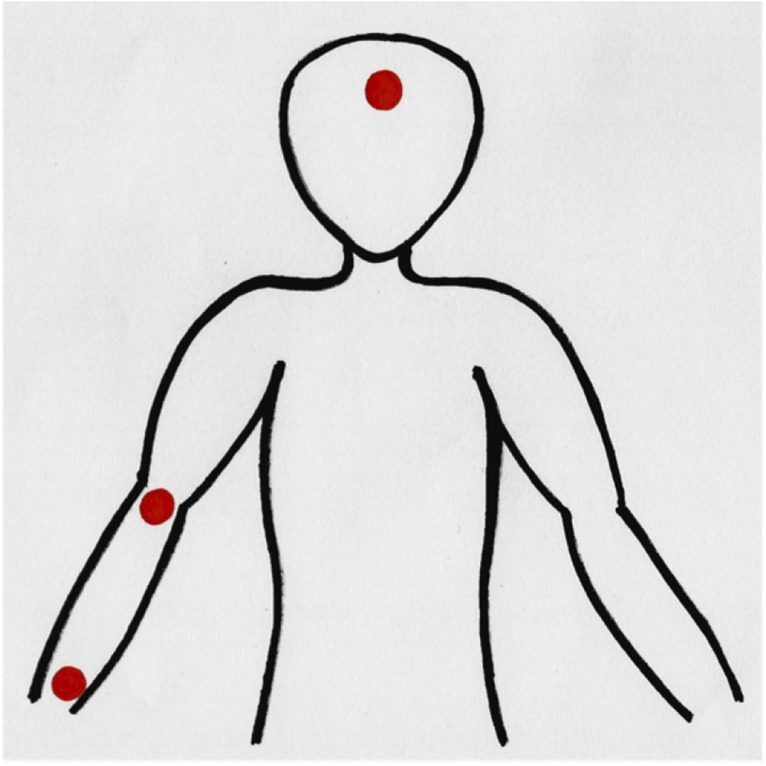
Sampling points (forehead, inside of the forearm distal and proximal).

**Figure 2:**
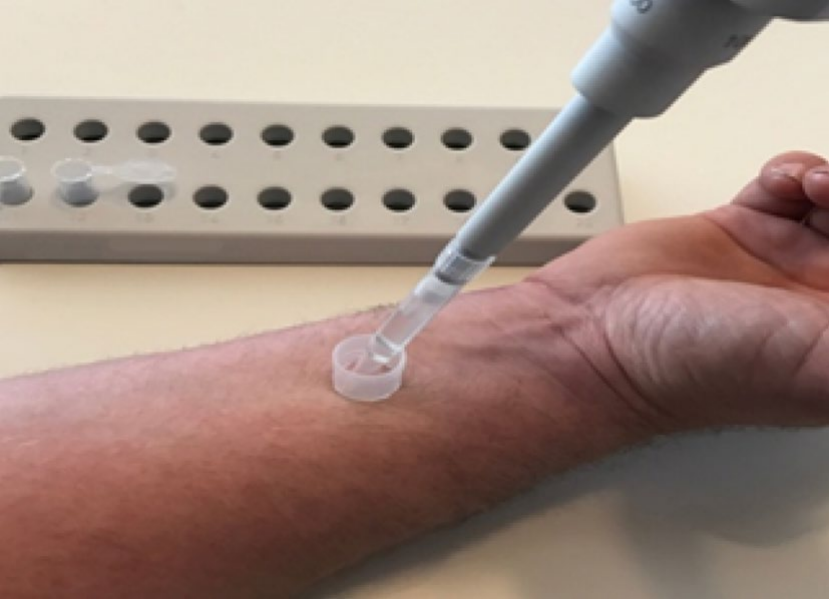
Sample collections from the inner side of the forearm (distal collection is shown); solvent is added and stirred.

The AMP concentrations on the skin surface were determined by a skin rinsing method as described elsewhere^21,22^. To this end standardized skin areas (1.77 cm^2^) from forehead and on the inside of the forearm (proximal and distal; see Figs. 1 and 2) were rinsed with 1 ml of a rinsing buffer solution (10 mM sodium phosphate buffer, pH 7.4 containing 0.1% Triton X-100) using a sterile plastic ring. The skin area was rinsed by pipetting ten times up and down. Subsequently, the skin rinsing solution was harvested, centrifuged (10 min, 10.000×*g*) and diluted 1:10 with rinsing buffer solution containing 10% (w/v) bovine serum albumin. The samples were stored at − 80 °C until further processing.

### Quantitative determination of the antimicrobial peptides by ELISA

Protein levels of psoriasin and RNase 7 in the skin washing fluids were measured using self-designed ELISAs, as described previously^23^. For the psoriasin ELISA, a monoclonal antibody from hybridoma mouse cells was used. The polyclonal antibody for the RNase 7 ELISA was derived from goat. To reach a high reliability, each sample was determined two times. The resulting double values were used to calculate a mean value for each sample.

### Additional measures

Socio-demographic characteristics and mental disorders were assessed on the basis of a clinical interview. Anthropometric measurements were used to allow for the calculation of BMI (kg/m^2^). All participants were weighed in their underwear using a standardized scale.

### Statistics

IBM SPSS Statistics Version 25 was used to perform the statistical analyses. Since most variables were not normally distributed, Mann–Whitney U tests were used to examine differences between independent groups (AN versus HC) and the Wilcoxon-test was used to analyze changes within the same group (patients with AN before and after weight gain). The medians and the interquartile ranges (IQR) were listed in addition to the mean values, the standard deviations and the range. The level of significance was set at *p* < 0.05.

In addition to significance testing, we calculated partial eta squared (η^2^) as effect size for non-parametric tests: 0.01 indicates a small effect, 0.06 a medium, and 0.14 a large effect^24^. Effect sizes are of importance in this study since the samples were small with large data variability. The sole use and often dichotomous interpretation of the *p* value has been repeatedly questioned. To prevent non-significant results from being falsely downgraded in their relevance, numerous authors recommend concentrating on effect sizes, since they do not depend on factors such as sample size^25,26^.

## Results

### Participants

Socio-demographic and weight related data are summarized in Table 1. In patients with AN, the mean duration of the eating disorder was 7.4 years (SD 8.2) with a range from 0.5 to 35.0 years. Between the first and the second testing, the mean BMI increased from 12.6 kg/m^2^ (SD 1.7) to 14.5 kg/m^2^ (SD 1.7), corresponding to a mean weight gain of 5.7 kg (SD 1.5) and an increase of 2.0 (SD 0.5) BMI points. In patients with AN we did not find any significant correlations between baseline AMP concentrations and age, illness duration, current BMI, lowest and highest lifetime BMI (Table 1 supplement). 24.2% (n = 8) of the patients with AN showed binge eating/purging behavior; 18.1% met criteria for a personality disorders, while 24.2% presented with obsessive–compulsive or anxiety disorders; 75.8% of the patients with AN were diagnosed with depression.

**Table 1:** Socio-demographic characteristics of patients with anorexia nervosa (AN) and healthy controls (HC).

	AN n = 33	HC n = 33
**Age at the time of first measurement**		
Mean (SD)	25.8 (10.0)	25.9 (10.1)
Median (IQR)	23.0 (8.0)	22.0 (6.0)
Range	17.0–54.0	16.0–57.0
**Partnership status,** % (n)		
Single	84.8 (28)	60.6 (20)
In partnership	15.2 (5)	39.4 (13)
**Educational level,** % (n)		
≥ 12 years of education	63.6 (21)	84.8 (28)
< 12 years of education	36.4 (12)	15.2 (5)
**Weight in kg, first measurement**		
Mean (SD)	35.4 (5.2)	61.5 (6.3)
Median (IQR)	36.9 (6.2)	61.0 (7.9)
Range	23.4–45.5	49.0–80.0
**Weight in kg, second measurement**		
Mean (SD)	40.8 (5.2)	–
Median (IQR)	42.4 (5.2)	–
Range	29.1–52.3	–
**BMI in kg/m**^**2**^**, first measurement**		
Mean (SD)	12.6 (1.7)	22.0 (1.8)
Median (IQR)	12.9 (3.1)	22.1 (2.6)
Range	9.2–14.8	18.7–25.8
**BMI in kg/m**^**2**^**, second measurement**		
Mean (SD)	14.5 (1.7)	–
Median (IQR)	14.8 (2.6)	–
Range	10.7–17.6	–
**Lowest lifetime weight in kg**		
Mean (SD)	32.3 (5.9)	56.5 (6.3)
Median (IQR)	33.5 (8.7)	56.9 (7.5)
Range	19.0–43.6	47.0–78.0
**Highest lifetime weight in kg**		
Mean (SD)	56.1 (12.1)	65.1 (7.9)
Median (IQR)	55.0 (9.3)	63.0 (10.5)
Range	36.0–110.0	52.5–82.0
**Lowest lifetime BMI in kg/m**^**2**^		
Mean (SD)	11.4 (1.8)	20.2 (1.7)
Median (IQR)	11.5 (2.7)	20.3 (2.3)
Range	7.9–14.6	17.6–25.0
**Highest lifetime BMI in kg/m**^**2**^		
Mean (SD)	20.0 (4.5)	23.3 (2.7)
Median (IQR)	19.1 (2.5)	23.1 (2.8)
Range	15.0–41.4	19.0–32.3

*AN* anorexia nervosa, *HC* healthy controls, *BMI* body mass index, *SD* standard deviation, *IQR* interquartile range.

### Comparison between patients with AN and healthy controls

Table 2 depicts the mean (SD) and median (IQR) values of the antimicrobial peptides psoriasin and RNase 7 on the three sampling sites. Overall, variances were high, psoriasin concentrations were higher compared to RNase 7 concentrations, and concentrations on the forehead were higher compared to the inner side of the forearm. The Mann–Whitney U test did not reveal statistically significant differences between patients with AN and healthy controls regarding AMP levels. However, by looking at the effect sizes, small effects for higher psoriasin and RNase 7 levels in healthy controls compared to participants with AN were found (psoriasin on the forehead η^2^ = 0.029, RNase 7 on the forehead η^2^ = 0.015, psoriasin on the distal forearm (η^2^ = 0.015) and RNase 7 on the proximal forearm (η^2^ = 0.018).

**Table 2:** Comparison of patients with anorexia nervosa (AN) and age- and sex-matched healthy controls (HC).

	AN n = 33	HC n = 33	Mann–Whitney U test
**Psoriasin (ng/ml) forehead**			U = 436.5
Mean (SD)	172.1 (155.5)	253.2 (264.0)	Z = − 1.385
Median (IQR)	146.8 (220.3)	181.2 (251.4)	*p* = 0.166
Range	1.6–664.6	23.8–1319.6	**η**^**2**^** = 0.029**
**Psoriasin (ng/ml) forearm proximal**			U = 527.2
Mean (SD)	10.1 (9.9)	7.4 (4.9)	Z = − 0.218
Median (IQR)	4.8 (14.4)	6.4 (6.5)	*p* = 0.827
Range	0.8–34.8	1.4–18.3	η^2^ = 0.001
**Psoriasin (ng/ml) forearm distal**			U = 466.0
Mean (SD)	6.0 (6.8)	8.9 (28.9)	Z = − 1.007
Median (IQR)	2.7 (6.1)	2.5 (4.0)	*p* = 0.314
Range	0.9–27.2	0.7–167.6	**η**^**2=**^**0.015**
**RNase 7 (ng/ml) forehead**			U = 466.5
Mean (SD)	2.1 (4.1)	1.8 (1.8)	Z = − 1.002
Median (IQR)	1.1 (1.2)	1.3 (1.3)	*p* = 0.316
Range	0.0–23.5	0.2–8.7	**η**^**2**^** = 0.015**
**RNase 7 (ng/ml) forearm proximal**			U = 459.0
Mean (SD)	1.2 (1.1)	1.2 (0.8)	Z = − 1.098
Median (IQR)	0.8 (0.8)	1.0 (0.9)	*p* = 0.272
Range	0.1–4.8	0.1–3.7	**η**^**2**^** = 0.018**
**RNase 7 (ng/ml) forearm distal**			U = 521.5
Mean (SD)	1.4 (1.1)	1.3 (1.0)	Z = − 0.296
Median (IQR)	0.9 (1.2)	1.0 (1.0)	*p* = 0.768
Range	0.3–4.6	0.1–4.9	η^2^ = 0.001

η^2^ eta squared, bold print indicates a small effect size.

*AN* anorexia nervosa, *SD* standard deviation, *IQR* interquartile range.

### Change in AMP levels after weight gain

The mean and median levels of psoriasin and RNase 7 on the three sampling points at the first and second measurement are displayed in Table 3. Psoriasin concentrations on the forehead increased significantly during weight gain with an intermediate effect size (*p* = 0.011, η^2^ = 0.101), while other differences did not reach statistical significance. However, by examining the effect sizes, we found some changes with a small effect, e.g. for psoriasin concentrations on the proximal and distal forearm (η^2^ = 0.012, η^2^ = 0.028). The AMP values at the second measurement also did not differ from the baseline values of the healthy controls (data not shown).

**Table 3:** Comparison between the first and the second measurement after weight gain in patients with anorexia nervosa (AN).

	First measurement n = 32	Second measurement n = 32	Wilcoxon test
**Psoriasin (ng/ml) forehead**			
Mean (SD)	173.5 (157.8)	273.9 (262.1)	Z = − 2.543
Median (IQR)	154.5 (232.7)	208.9 (328.3)	*p* = 0.011
Range	1.6–664.6	2.9–1144.7	**η**^**2**^** = 0.101**
**Psoriasin (ng/ml) forearm proximal**			
Mean (SD)	10.4 (10.0)	6.9 (5.5)	Z = − 1.328
Median (IQR)	5.4 (14.4)	4.8 (6.0)	*p* = 0.184,
Range	0.8–34.8	1.0–26.5	**η**^**2**^** = 0.028**
**Psoriasin (ng/ml) forearm distal**			
Mean (SD)	6.1 (6.8)	6.3 (5.2)	Z = − 0.882
Median (IQR)	3.1 (6.5)	4.7 (6.5)	*p* = 0.378
Range	0.9–27.2	0.6–17.4	**η**^**2**^** = 0.012**
**RNase 7 (ng/ml) forehead**			
Mean (SD)	2.2 (4.2)	2.2 (3.0)	Z = − 1.119
Median (IQR)	1.1 (1.1)	1.1 (1.3)	*p* = 0.263
Range	0.0–23.5	0.2–14.3	**η**^**2**^** = 0.02**
**RNase 7 (ng/ml) forearm proximal**			
Mean (SD)	1.1 (1.2)	1.2 (1.1)	Z = − 0.541
Median (IQR)	0.8 (0.7)	0.9 (1.0)	*p* = 0.588
Range	0.1–4.8	0.0–3.9	η^2^ = 0.005
**RNase 7 (ng/ml) forearm distal**			
Mean (SD)	1.4 (1.1)	1.8 (2.7)	Z = − 0.078
Median (IQR)	1.0 (1.2)	1.1 (1.8)	*p* = 0.937
Range	0.3–4.6	0.0–15.2	η^2^ = 0.0

η^2^ eta squared, bold print indicates a small to moderate effect size.

*SD* standard deviation, *IQR* interquartile range.

## Discussion

This is the first study to assess AMP expression in healthy skin of patients with AN from skin washing fluids. Contrary to our hypothesis which was based on results in *Drosophila melanogaster*, expression of the AMPs psoriasin and RNase 7 in the skin did not differ significantly between patients with severe AN (BMI < 15 kg/m^2^) and HC. Above significance, HC even had higher AMP levels compared to AN with a small effect size. Additionally, AMP expression increased with weight regain, which was significant only for psoriasin on the forehead with a medium effect size.

The AMPs psoriasin and RNase 7 in human skin do not appear to contribute to the low incidence of infections described in patients with AN. However, we measured only two of a large number of AMPs and only AMPs secreted in the human skin but not in the respiratory or gastrointestinal system. The increase of the AMP psoriasin upon weight gain might be explained by an increase of cells of the lipid-rich sebaceous glands. It is known that large amounts of psoriasin can be found in lipid extracts of sebaceous gland–rich scalp skin as opposed to only small amounts of psoriasin in washing fluids of the forearm skin^21^. In AN substantial dry skin (asteatotic skin, xerosis) of the whole body, including the scalp hair, is frequently found with sebum production reported to be decreased on average by 40%^27^. However, considering that the mean weight gain was only 5.7 kg (BMI increase 2.0 kg/m^2^) and that the participants were still markedly underweight at the second measurement point, the increase of psoriasin secretion on the forehead seems remarkable. Overall, also non-pathogen factors can induce AMP expression (calcium, zinc, albumin, butyrate, lactose, vitamin D3); thus, improved nutrition during weight restitution in patients with AN might contribute to an increase of AMP expression^28^.

Alternatively, it is possible that patients with AN show low rates of symptomatic viral and bacterial infections but higher rates of asymptomatic or subclinical infections. There is evidence from retrospective chart reviews that patients with AN with bacterial infections show reduced fever response and fewer signs and symptoms and might experience a delay in diagnosis^29^. Thus, rates of infections might be underestimated. This assumption would fit our finding of low AMP suppression.

Overall, our results are preliminary, difficult to interpret and they raise further questions. It has to be considered that a starvation-regulated induction of RNase 7 and psoriasin in human skin cells might not exist. Alternatively, undernutrition in patients with AN does not necessarily lead to protein deficiency^3,4,7^, so the observed results in starving *Drosophila melanogaster* might not be easily transferable to the nutritional status of patients with AN. Finally, the AMPs measured in our study might react differently compared to AMPs produced in airway epithelia, the gastrointestinal or urogenital tract. Further, an increase of cytokine levels in patients with AN has only been reported in serum or plasma^30^, so there is no information about the concentrations of local pro-inflammatory cytokine levels in skin.

Our study has several limitations. Firstly, we analyzed only two AMPs due to methodological availability. Moreover, we considered only constitutive expression; no microbial stimulation was conducted. Since this study is limited to AMPs on the skin surface, we cannot draw any conclusions on the AMP levels in other tissues. Further, we did not measure cytokines or other immunological parameters that have shown to be changed in patients with AN and that have shown to influence AMP secretion. Healthy human skin is characterized by the expression of AMPs in variable amounts. Large intra- and interindividual variabilities have been noted^22^ which increases the likelihood of a Type II error in addition to our small sample size.

Despite these limitations, our study achieved reliable results. This is attributable to the use of narrow inclusion criteria which led to a homogenous group of patients with AN. Only patients with a BMI lower than 15 kg/m^2^ were included. Secondly, weight gain was verified using bioelectrical impedance analyses prior to the second testing to differentiate water retention from the actual gain of cell mass. Another strength of this study is the high reproducibility. This was achieved by following precise procedures for each sample collection^23^. Further, sample collection was always carried out in the same room with strong hygienic guidelines. To maximize experimental reliability, double values were used for each sample in the ELISA.

In summary, we found that patients with AN exhibited somewhat lower but non-significant concentrations of AMPs (psoriasin, RNase 7) on the skin with small effect sizes and that their concentration increased with weight gain. Future studies should include a larger set of AMPs of different cellular sources and different tissues including a larger sample number. Long-term studies seem interesting since our short-term observation with a mean weight gain of only 5.7 kg (2.0 kg/m^2^) delivered a remarkable increase in AMP levels at least on the forehead. It is also an interesting question whether changes in AMP expression may influence body weight. To conclude, our study contributes to the knowledge of alterations in the immune system of AN patients.

## Supplementary information

Supplementary Table 1.
