# Supplementary material for: Antimicrobial peptides in patients with anorexia nervosa: comparison with healthy controls and the impact of weight gain
Source: Sci Rep. 2020 Dec 17;10:22223. doi: 10.1038/s41598-020-79302-1 (PMC7746688; doi:10.1038/s41598-020-79302-1)
Supplement: Supplementary file 1 — Supplementary Table 1. [file 41598_2020_79302_MOESM1_ESM.pdf]

Antimicrobial peptides in patients with anorexia nervosa: comparison with healthy controls and the impact of weight gain

Marie-Christin Bendix, Michael Stephan, Mariel Nöhre, Wally Wünsch-Leiteritz, Hagen Schmidt, Gisa Tiegs, Jürgen Harder, Martina de Zwaan

Table 1 supplement. Pearson’s correlations between baseline AMP concentrations in patients with AN with age, illness duration, current BMI, lowest and highest lifetime BMI.

	**Age**	**Illness duration**	**Current BMI**	**Lowest BMI**	**Highest BMI**
**Psoriasin (ng/ml) forehead**					
r	-.095	-.189	.169	.085	.118
p	.599	.291	.348	.638	.513
**Psoriasin (ng/ml) forearm**					
**proximal**	.266	.311	.079	.008	.110
r	.134	.078	.661	.964	.542
p					
**Psoriasin (ng/ml) forearm distal**					
r	.265	.100	-.202	-.222	.021
p	.136	.579	.259	.215	.906
**RNase 7 (ng/ml) forehead**					
r	.028	-.144	.169	.085	.141
p	.875	.422	.348	.638	.434
**RNase 7 (ng/ml) forearm proximal**					
r	-.056	-.091	.212	.211	.044
p	.756	.614	.236	.238	.808
**RNase 7 (ng/ml) forearm distal**					
r	-.045	-.062	.271	.137	.138
p	.805	.730	.128	.447	.444

r = correlation coefficient
